# Factors Related to a Right-Left Difference in Visual Field Defect in the Eyes with Untreated Normal Tension Glaucoma

**DOI:** 10.1155/2018/4595214

**Published:** 2018-02-15

**Authors:** Haruka Moroi, Ayako Anraku, Kyoko Ishida, Goji Tomita

**Affiliations:** ^1^Department of Ophthalmology, Toho University Ohashi Medical Center, 2-17-6 Ohashi, Meguro-ku, Tokyo 153-8515, Japan; ^2^Department of Ophthalmology, Kanto Rosai Hospital, 1-1 Kigetsu Sumiyoshi-cho, Nakahara-ku, Kawasaki 211-8510, Japan

## Abstract

**Purpose:**

To investigate factors related to a right-left difference in visual field defect in untreated normal tension glaucoma (NTG).

**Methods:**

The medical records of 92 patients with untreated NTG were reviewed. Ocular blood flow was evaluated with laser speckle flowgraphy, and the mean blur rate (MBR) at the optic nerve head was analyzed. Relationships between right-left differences in mean deviation (MD), intraocular pressure, MBR, spherical equivalent, central corneal thickness, and mean ocular perfusion pressure were evaluated using Spearman's rank correlation coefficient. Multiple regression analysis was used to detect factors contributing to a right-left difference in MD.

**Results:**

The right-left difference in MD was correlated with differences in intraocular pressure (*r* = −0.263, *P* = 0.011), MBR (*r* = 0.417, *P* < 0.001), and spherical equivalent (*r* = 0.213, *P* = 0.042), but not with central corneal thickness or mean ocular perfusion pressure. Multiple regression analysis showed that a difference in MBR was the only significant contributor to a right-left difference in MD (slope 0.047, 95% confidence interval 0.025–0.069; *P* < 0.001).

**Conclusion:**

In untreated NTG, a difference in blood flow at the optic nerve head was a significant contributor to a right-left difference in visual field defect.

## 1. Introduction

Previous studies have reported a correspondence between intraocular pressure (IOP) and greater visual damage when the IOP is not equal between the right and left eyes in patients with normal tension glaucoma (NTG) [1–4]. However, the randomized controlled Low-Pressure Glaucoma Treatment Study of 190 patients with NTG reported that asymmetric IOP was not related to visual field asymmetry [5]. It has also been reported that about 70% of patients with asymmetric visual field defects do not have unequal mean IOP [1, 2]. Therefore, factors other than IOP are likely to contribute to development of visual field defects in patients with NTG.

Lee et al. reported that patients with NTG and asymmetric progression showed a decrease in the diameter of the central retinal artery over time in the eye that progressed but not in the eye that remained stable [6]. Further, in a study of retrobulbar hemodynamics in patients with NTG who had asymmetric visual field change and asymmetric ocular perfusion pressure, Kondo et al. found that the flow velocity and resistance index in the ophthalmic artery were significantly higher in subjects with higher ocular perfusion pressure in the eye that had a worse mean deviation (MD) than in subjects with higher ocular perfusion pressure in the eye that had better MD [7]. These results suggest that vascular abnormalities are involved in the development and progression of NTG.

Laser speckle flowgraphy (LSFG) utilizes the laser speckle phenomenon to measure ocular blood flow in a noninvasive manner [8]. LSFG provides the mean blur rate (MBR), a relative measure of blood flow expressed in arbitrary units (AU). Although MBR is not an exact measure, it is proportional to blood velocity and has been used to measure relative differences in blood flow at the optic nerve head (ONH) [9–12]. The MBR of the ONH has been shown to be strongly correlated with absolute blood flow values measured by hydrogen gas clearance or the microsphere method in primates and rabbits [13–15]. Moreover, LSFG was reported to be a reliable method for quantitative assessment of ocular blood flow in Japanese [16] and west European populations [17].

There has been one study that investigated intraindividual (right-left) correlations between the microcirculation at the ONH as measured by scanning laser Doppler flowmetry and visual field parameters in patients with treated primary open-angle glaucoma (POAG) [18]. However, to the best of our knowledge, there have been no reports on the association of an interocular difference in ONH blood flow with a right-left difference in visual field defect in untreated NTG. Therefore, we investigated the factors associated with a right-left difference in visual field defect in untreated NTG using LSFG.

## 2. Materials and Methods

### 2.1. Study Subjects

The study protocol was approved by the Toho University Ohashi Medical Center Institutional Review Board (approval number H16061) and adhered to the tenets of the Declaration of Helsinki. We retrospectively reviewed the medical records of patients with glaucoma in whom the ocular circulation was measured by LSFG before initiation of glaucoma treatment at the Toho University Ohashi Medical Center between January 2013 and June 2016. Subjects who met all of the following criteria were included: untreated NTG with normal and open anterior chamber angles on slit-lamp biomicroscopy and gonioscopy, an ONH that appeared to be glaucomatous on stereoscopic evaluation with a corresponding visual field defect, IOP ≤ 21 mmHg throughout 1 day (measured every 3 hours) or on at least three separate days, a spherical refractive error between −8.00 and +4.00 diopters (D), a cylindrical refractive error within 2.5 D, and best-corrected visual acuity of at least 20/25. A visual field defect was defined as having three or more significant (*P* < 0.05) nonedge-contiguous points, with at least one point at the *P* < 0.01 level in the pattern deviation plot, along with grading outside normal limits on the glaucoma hemifield test. Subjects were excluded from the analysis if they had significant cataract which may influence refractive errors and visual fields, a history of intraocular surgery, intraocular disease (other than NTG), or systemic disease that could affect visual field test results. The IOP was measured with a Goldmann applanation tonometer, and the mean IOP for three separate days was used for the analyses.

Systolic blood pressure (SBP) and diastolic blood pressure (DBP) were measured before performing the LSFG measurements. The mean blood pressure (MBP) and mean ocular perfusion pressure (MOPP) were calculated as follows:

(1) $$\begin{array}{c} MBP=DBP+\frac{1}{3}\left(SBP-DBP\right),\\ MOPP=\frac{2}{3}\left(\;MBP-IOP\right). \end{array}$$

### 2.2. Laser Speckle Flowgraphy

Blood flow at the ONH was evaluated with LSFG (LSFG-NAVI version 3.1.39.2 software, Softcare Ltd., Fukuoka, Japan), and the MBR was used as a relative measure of blood flow. The principle and methods of LSFG have been described elsewhere [8, 19]. Briefly, the instrument is comprised of a fundus camera equipped with a diode laser (wavelength 830 nm) and a digital charge-coupled device camera (resolution 750 × 360 pixels). The ONH margins were measured with an ellipsoidal band, and the position of the ONH was saved in the system software. The LSFG analysis software automatically calculated the mean MBR in all areas of the optic disc, the mean MBR in the vessel area of the optic disc, and the mean MBR in the tissue area of the optic disc. We used the mean of the MBR values in all areas of the optic disc in this study. The pupils of the eyes enrolled in the study were dilated using 0.4% tropicamide before LSFG examination, and three consecutive measurements were taken for each subject. The average of the three measurements was used in the analyses.

### 2.3. Visual Field Analyses

Standard automated perimetry was performed using a Humphrey Field Analyzer (Carl Zeiss Meditec Inc., Dublin, CA, USA) with the 30-2 Swedish Interactive Threshold Algorithm. Visual field tests were considered reliable when fixation losses were <20%, false positives were <15%, and false negatives were <25%. For subset analysis, the study eyes were allocated according to their MD value and mean IOP to an IOP-MD-concordant group (higher mean IOP in the eyes with worse MD) and an IOP-MD-discordant group (equal or lower mean IOP in the eyes with worse MD). Better MD and worse MD were defined according to the MD values of the visual field test performed within 3 months of the LSFG measurements. Two or more examinations were required to confirm a difference in MD between the eyes if the interocular difference was within 2 dB. The dB values were converted to the 1/Lambert linear scale for analysis.

### 2.4. Statistical Analysis

The Wilcoxon signed-rank test was used to evaluate differences between the eyes in each group of subjects. The Mann–Whitney *U* test was for the evaluation of interocular differences between the groups. Categorical data were compared using the chi-square test. Spearman's rank correlation coefficients were calculated to evaluate the relationship between right-left differences in ocular parameters. Multiple regression analysis was used to detect factors contributing to a right-left difference in the visual field defect, that is, age, sex, IOP, spherical equivalent (SE), MBR, central corneal thickness (CCT), and MOPP. The data are reported as the mean ± standard deviation. A *P* value < 0.05 was considered to be statistically significant.

## 3. Results

Ninety-two subjects (34 male, 58 female, mean age 54.1 ± 11.9 y) with untreated NTG were included in the study. The demographic and clinical characteristics of subjects are shown in Table 1. The eye with higher IOP had worse MD in 47 (51%) of the subjects, and the eye with a lower MBR had worse MD in 62 (67%). A comparison of ocular parameters between the eyes with worse MD and those with better MD is provided in Table 2. The eyes with worse MD were more myopic than those with better MD (mean SE −3.77 ± 3.1 D versus −3.48 ± 3.0, *P* = 0.011). Although there was no significant difference in mean IOP between the eyes with worse MD and those with better MD (14.8 ± 2.3 mmHg versus 14.7 ± 2.3 mmHg, *P* = 0.051), the MBR was significantly lower in the eyes with worse MD than in those with better MD (18.4 ± 4.1 AU versus 19.5 ± 4.8 AU, *P* = 0.001).

**Figure 1:**
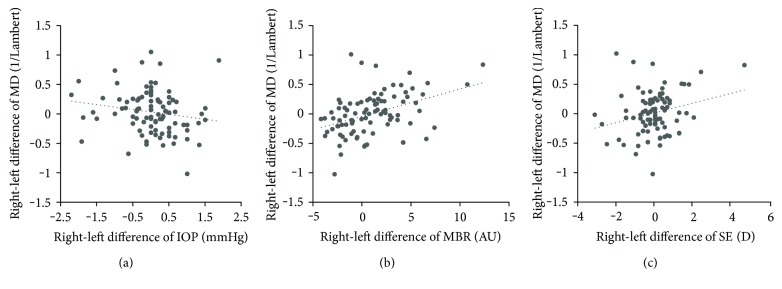
Scatterplots of right-left difference in MD versus right-left difference in IOP (a), MBR (b), and SE (c) in all 92 subjects. IOP, intraocular pressure; MBR, mean blur rate; MD, mean deviation; and SE, spherical equivalent.

Correlations between the right-left difference in MD and the ocular parameters are shown in Table 3. The right-left difference in MD had significant correlations with the difference in mean IOP (*r* = −0.263, *P* = 0.011), MBR (*r* = 0.417, *P* < 0.001), and SE (*r* = 0.213, *P* = 0.042), but not with the difference in CCT (*r* = 0.160, *P* = 0.129) or MOPP (*r* = 0.190, *P* = 0.070). The scatterplots comparing the right-left difference in MD with the differences in mean IOP, MBR, and SE are shown in Figure 1.

Multiple regression analysis for all 92 subjects, wherein the right-left difference in MD was used as the dependent variable and age, sex, intraocular differences in MBR, and mean IOP, SE, CCT, and MOPP were used as explanatory variables, showed that an intraocular difference in MBR was a significant contributor to the right-left difference in MD (slope 0.047, *β* = 0.413, 95% confidence interval (CI) 0.025–0.069; *P* < 0.001).

When the subjects were divided into groups according to MD and mean IOP for subset analysis, 47 (51%) of the 92 subjects were in the IOP-MD-concordant group (higher mean IOP in the eye with worse MD) and 45 (49%) were in the IOP-MD-discordant group (equal or lower mean IOP in the eye with worse MD).  Table 4 shows a comparison of the demographic characteristics and interindividual differences in ocular parameters between the two groups. In the IOP-MD-concordant group, the values for age (56.5 ± 12.5 y versus 51.3 ± 11.0 y; *P* = 0.027) and absolute right-left difference in mean IOP (0.67 ± 0.51 mmHg versus 0.42 ± 0.53 mmHg; *P* = 0.002) were greater than in the IOP-MD-discordant group.

**Figure 2:**
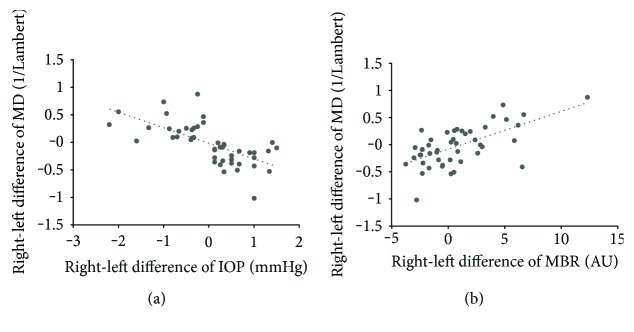
Scatterplots of right-left difference in MD versus right-left difference in IOP (a) and MBR (b) in the IOP-MD-concordant group (*n* = 47). IOP, intraocular pressure; MBR, mean blur rate; and MD, mean deviation.

**Figure 3:**
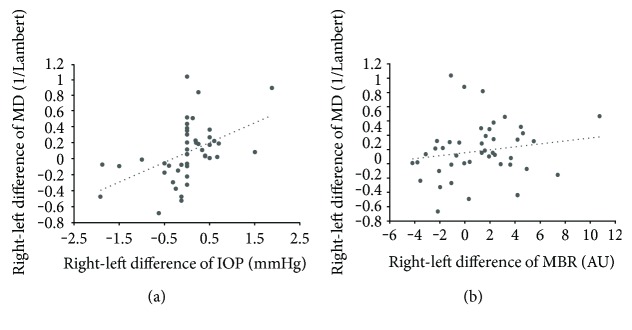
Scatterplots of right-left difference in MD versus right-left difference in IOP (a) and MBR (b) in the IOP-MD-discordant group (*n* = 45). IOP, intraocular pressure; MBR, mean blur rate; and MD, mean deviation.

In the IOP-MD-concordant group (*n* = 47), the right-left difference in MD was significantly correlated with the differences in mean IOP (*r* = −0.763, *P* < 0.001) and MBR (*r* = 0.563, *P* < 0.001) but not with the difference in SE (*r* = 0.225, *P* = 0.129).  Figure 2 shows the scatterplots comparing the right-left difference in MD with the differences in mean IOP and MBR in the IOP-MD-concordant group. In the IOP-MD-discordant group (*n* = 45), the right-left difference in MD was significantly correlated with the difference in mean IOP (*r* = 0.602, *P* < 0.001) but not with the difference in MBR (*r* = 0.187, *P* = 0.219) or SE (*r* = 0.165, *P* = 0.279).  Figure 3 shows the scatterplots comparing the right-left difference in MD with the differences in mean IOP and MBR in the IOP-MD-discordant group.

The multiple regression analysis that included subjects in the IOP-MD-concordant group (*n* = 47), wherein the right-left difference in MD was used as the dependent variable and age, sex, intraocular differences in MBR, and mean IOP, SE, CCT, and MOPP were used as explanatory variables, showed that the intraocular difference in MBR (slope 0.048, *β* = 0.437, 95% CI 0.026–0.071; *P* < 0.001) and mean IOP (slope −0.211, *β* = −0.500, 95% CI −0.298–0.124; *P* < 0.001) contributed significantly to the right-left difference in MD. In contrast, the multiple regression analysis that included subjects in the IOP-MD-discordant group (*n* = 45) and the same variables found that only the intraocular difference in mean IOP contributed significantly to the right-left difference in MD (slope 0.250, *β* = 0.476, 95% CI 0.108–0.392; *P* = 0.001).

## 4. Discussion

Glaucoma is considered to be a multifactorial disease and is associated with a combination of IOP-dependent and IOP-independent risk factors, including decreased ocular blood flow [10, 20]. Recently, Shiga et al. reported that blood flow at the ONH was significantly reduced in patients with preperimetric glaucoma (PPG) when compared with that in normal subjects [11]. Their finding is consistent with that in an earlier study using optical coherence tomography (OCT) angiography that reported perfusion of the ONH to be significantly lower in patients with PPG than in normal subjects [21]. These findings indicate that blood flow at the ONH is impaired in the very early stages of glaucoma.

The present study identified a significant correlation between a right-left difference in ONH blood flow and a difference in the visual field defect in patients with untreated NTG. This result is consistent with that of Ciancaglini et al. [18], who reported that side differences in visual field index were significantly correlated with side differences in the vascular parameters of the lamina cribrosa in treated patients with POAG. Moreover, previous studies have reported that patients with asymmetric glaucomatous visual field loss show asymmetric flow velocities in the central retinal artery and ophthalmic artery [7, 22, 23]. These findings suggest that the pathogenesis of the disease may include vascular abnormalities.

In this study, 51% of eyes with higher IOP had greater visual field damage, that is, the IOP-MD-concordant group. The subjects in this group were significantly older than those in the IOP-MD-discordant group, and the absolute difference in mean IOP in the IOP-MD-concordant group was greater than that in the IOP-MD-discordant group. These results suggest that advancing age and a greater difference in IOP are associated with asymmetric visual field loss in some patients. Our results are consistent with a previous report showing that 53 (60%) of 88 patients with NTG had more severe visual field defects in the eye with higher IOP than in the fellow eye with lower IOP [4]. However, in the multiple regression analysis that included all subjects, the intraocular difference in IOP was not a significant contributor to the right-left difference in MD. In this study, the mean IOP values recorded on three separate days were used for the analysis and circadian variation in IOP was not evaluated. It is known that variability in nocturnal IOP is an important clinical determinant of the likelihood of progression of visual field defects [24]. Moreover, Kiuchi et al. reported that visual field damage was more severe in eyes with a greater magnitude of IOP elevation in response to postural changes [25]. Besides, some reports showed that IOP elevation asymmetry in lateral decubitus position was associated with asymmetric visual field damage [26–29]. Therefore, it is possible that the mean of IOP values recorded on three separate days may have been insufficient to assess the intraocular difference in IOP.

In the IOP-MD-discordant group, eyes with more visual field damage tended to have lower IOP. We suspected that impaired blood flow at the ONH might cause this inverse relationship. However, MBR did not contribute to the difference in MD in this group. Previous studies reported that right-left differences in axial length and disc area were associated with a right-left difference in the visual field defect in patients with NTG [30, 31]. Further, a recent study using swept-source OCT found that the lamina cribrosa was thinner in patients with PPG than in normal subjects [32] and reported that the thickness of the lamina cribrosa was significantly correlated not only with MD but also with the vertical cup-disc ratio. These results suggest that a structural change in the optic nerve contributes to asymmetry of the visual field. In the present study, there was a tendency for the eyes with worse MD to be more myopic than those with better MD. However, we did not evaluate morphologic changes in the ONH in all subjects. When we analyzed the data in 60 patients who had OCT data of good quality and matched between structural and functional losses (data are not shown), multiple regression analysis showed that intraocular difference in MBR (slope 0.059, *β* = 0.489, 95% CI 0.034–0.084; *P* < 0.001) and SE (slope 0.111, *β* = 0.338, 95% CI 0.043–0.178; *P* = 0.002) contributed significantly to the right-left difference in MD.

It has also been reported that asymmetric CCT is associated with visual field asymmetry in patients with POAG [33]. However, in a more recent report by the same group, asymmetric POAG was associated with asymmetric dynamic contour tonometry but not with CCT [34]. Another study reported that visual field asymmetry in POAG was associated with corneal hysteresis but not with CCT [35]. In the present study, an intraocular difference in CCT did not contribute significantly to a right-left difference in MD.

Our study has several limitations. First, its retrospective nature may have introduced a degree of selection bias. Second, as mentioned earlier, we did not evaluate circadian variation in IOP, or effects of postural changes on IOP, so our assessment of the intraocular difference in IOP may have been incomplete. Third, we did not exclude the patients with systemic diseases such as diabetes mellitus, hypertension, or cardiovascular disease. Those systemic diseases may cause asymmetric insufficiency in ocular blood flow, which might have influenced on our results. However, when we reanalyzed factors related to a right-left difference in visual field defects in 74 patients without systemic disease, MBR was still detected as the only significant contributor (slope 0.047, *β* = 0.402, 95% CI 0.022–0.072; *P* < 0.001). Fourth, this study included subjects whose interocular difference in MD was within 2 dB. Although we confirmed the difference in MD between the right eye and left eye on more than two examinations, a small interocular MD difference may be affected by fluctuation of visual fields [36]. Since our subjects were newly diagnosed and untreated NTG patients, subjects with relatively earlier stage of glaucoma were included. It may cause mismatches between structural and functional damage. Assessment of subjects with greater MD asymmetry might have led to different results. However, subjects with an interocular difference in MD of more than 2 dB (data not shown) in multiple regression analysis showed that an intraocular difference in MBR contributed significantly to the right-left difference in MD (slope 0.060, *β* = 0.457, 95% CI 0.025–0.095; *P* = 0.001). Finally, in this study, we did not evaluate axial lengths, morphologic changes in the ONH, or structural parameters such as retinal nerve fiber layer. In subanalysis for 60 patients who had OCT data and matched between structural and functional losses, multiple regression analysis showed that intraocular difference in MBR (slope 0.059, *β* = 0.489, 95% CI 0.034–0.084; *P* < 0.001) and SE (slope 0.111, *β* = 0.338, 95% CI 0.043–0.178; *P* = 0.002) contributed significantly to the right-left difference in MD. Recruitments of more subjects with greater MD asymmetry and assessment of structural damage are needed as a further research.

## 5. Conclusion

In this study, we demonstrated that a difference in blood flow at the ONH contributed significantly to the right-left difference in visual field defect in patients with untreated NTG. This suggests that eyes with more visual field damage have a greater reduction in ONH blood flow. It is uncertain whether the decrease in ONH blood flow is a primary or secondary event caused by glaucomatous optic neuropathy; however, impaired ONH blood flow has a possible role in the pathogenesis of NTG.

## Figures and Tables

**Table 1:** Demographics and clinical characteristics of subjects.

Demographic characteristics	
Age (years)	54.1 ± 11.9
Sex (male/female)	34/58
Clinical characteristics	
SBP (mmHg)	122.0 ± 16.1
DBP (mmHg)	71.3 ± 12.5
MBP (mmHg)	88.2 ± 13.0
Diabetes mellitus	1 (1%)
Hypertension	16 (17%)
Cardiovascular disease	6 (7%)

The data are given as mean ± SD; SBP: systolic blood pressure; DBP: diastolic blood pressure; MBP: mean blood pressure.

**Table 2:** Comparison of ocular parameters between the eyes with worse MD and the eyes with better MD (*n* = 92).

	Eyes with worse MD	Eyes with better MD	*P* value
Mean IOP (mmHg)	14.8 ± 2.3	14.7 ± 2.3	0.051
SE (diopters)	−3.77 ± 3.1	−3.48 ± 3.0	**0.011**
MD (dB)	−5.20 ± 5.2	−2.22 ± 3.7	**<0.001**
PSD (dB)	7.52 ± 4.5	3.85 ± 3.4	**<0.001**
MBR (AU)	18.4 ± 4.1	19.5 ± 4.8	**0.001**
MOPP (mmHg)	43.6 ± 8.6	43.7 ± 8.6	0.168
CCT (*μ*m)	526.8 ± 31.3	528.3 ± 31.5	0.194

The data are given as mean ± SD; values in bold are statistically significant (*P* < 0.05). AU: arbitrary units; CCT: central corneal thickness; IOP: intraocular pressure; MBR: mean blur rate; MD: mean deviation; MOPP: mean ocular perfusion pressure; PSD: pattern standard deviation; SE: spherical equivalent.

**Table 3:** Correlation between the right-left difference in mean deviation and ocular parameters (*n* = 92).

	Difference in MD *r*	*P* value
Difference in mean IOP	**−0.263**	**0.011**
Difference in SE	**0.213**	**0.042**
Difference in MBR	**0.417**	**<0.001**
Difference in CCT	0.160	0.129
Difference in MOPP	0.190	0.070

Values in bold are statistically significant (*P* < 0.05). CCT: central corneal thickness; IOP: intraocular pressure; MBR: mean blur rate; MD: mean deviation; MOPP: mean ocular perfusion pressure; SE: spherical equivalent; *r*: Spearman's rank correlation coefficient.

**Table 4:** Comparison of the IOP-MD-concordant group and IOP-MD-discordant group.

	IOP-MD-concordant group (*n* = 47)	IOP-MD-discordant group (*n* = 45)	*P* value
Age (years)	**56.5 ± 12.5**	**51.3 ± 11.0**	**0.027**
Sex (male/female)	15/32	19/26	0.306
MBP (mmHg)	87.0 ± 13.4	89.3 ± 12.5	0.456
Absolute difference in mean IOP (mmHg)	**0.67 ± 0.51**	**0.42 ± 0.53**	**0.002**
Absolute difference in SE (diopters)	0.68 ± 0.69	0.78 ± 0.86	0.449
Absolute difference in MD (dB)	3.15 ± 3.11	2.80 ± 2.61	0.648
Absolute difference in PSD (dB)	4.02 ± 3.45	3.54 ± 3.39	0.401
Absolute difference in MBR (AU)	2.45 ± 2.27	2.63 ± 1.99	0.451
Absolute difference in CCT (*μ*m)	8.57 ± 8.96	9.07 ± 9.46	0.916

Values in bold are statistically significant. AU: arbitrary units; CCT: central corneal thickness; IOP: intraocular pressure; MBP: mean blood pressure; MBR: mean blur rate; MD: mean deviation; PSD: pattern standard deviation; SE: spherical equivalent.
